# Synthetic immunity by remote control

**DOI:** 10.7150/thno.41305

**Published:** 2020-02-19

**Authors:** Lena Gamboa, Ali H. Zamat, Gabriel A. Kwong

**Affiliations:** 1 The Wallace H. Coulter Department of Biomedical Engineering, Georgia Institute of Technology & Emory University, Atlanta, GA 30332, USA; 2 Institute for Electronics and Nanotechnology, Georgia Institute of Technology, Atlanta, GA 30332, USA; 3 Parker H. Petit Institute of Bioengineering and Bioscience, Georgia Institute of Technology, Atlanta, GA 30332, USA; 4 Integrated Cancer Research Center, Georgia Institute of Technology, Atlanta, GA 30332, USA; 5 Georgia Immunoengineering Consortium, Emory University and Georgia Institute of Technology, Atlanta, GA 30332, USA

**Keywords:** remote control, synthetic immunity, engineered cells, gene switches, immunotherapy

## Abstract

Cell-based immunotherapies, such as T cells engineered with chimeric antigen receptors (CARs), have the potential to cure patients of disease otherwise refractory to conventional treatments. Early-on-treatment and long-term durability of patient responses depend critically on the ability to control the potency of adoptively transferred T cells, as overactivation can lead to complications like cytokine release syndrome, and immunosuppression can result in ineffective responses to therapy. Drugs or biologics (e.g., cytokines) that modulate immune activity are limited by mass transport barriers that reduce the local effective drug concentration, and lack site or target cell specificity that results in toxicity. Emerging technologies that enable site-targeted, remote control of key T cell functions - including proliferation, antigen-sensing, and target-cell killing - have the potential to increase treatment precision and safety profile. These technologies are broadly applicable to other immune cells to expand immune cell therapies across many cancers and diseases. In this review, we highlight the opportunities, challenges and the current state-of-the-art for remote control of synthetic immunity.

## Introduction

Genetically reprogrammed immune cells have led to tremendous clinical success. T cells, natural killer (NK) cells and monocytes have been engineered to target tumors, eliminate infectious pathogens, and promote tissue regeneration 1, 2. Adoptive transfer of engineered T cells bearing chimeric antigen receptors (CARs) that redirect T cell cytotoxicity toward cancer cells, for instance, has shown remarkable efficacy in treating B cell malignancies 3. This success has inspired the use of immune cell therapies in other clinical settings, where some have even emerged as viable treatments for autoimmune disorders such as allergy and lupus 4, 5. In treating solid tumors, however, several challenges remain in achieving an effective antitumor response. These include lack of unique and targetable tumor antigens, low persistence and durability of engineered cells, inefficient trafficking and infiltration of cytotoxic T cells into the tumor microenvironment, and immunosuppression by the tumor microenvironment. Biologics designed to enhance T cell activity (e.g., IL-2) or block inhibitory signals (e.g., αPD-1) affect both adoptively transferred and endogenous cell populations, which can result in systemic toxicities including off-target cell killing. Additional challenges persist in mitigating adverse effects associated with strong anti-tumor responses, including cytokine release syndrome, neurologic toxicity, “on-target, off-tumor” killing, and anaphylaxis 6.

**Figure 1:**
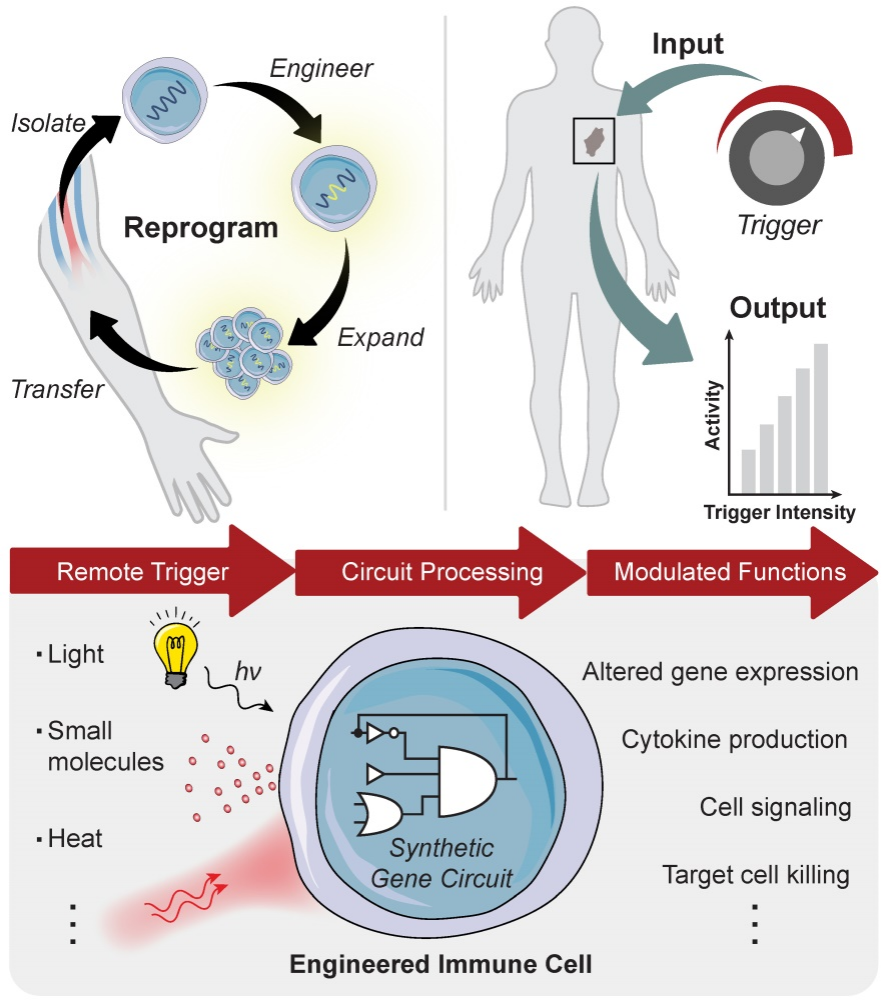
** Remote control of immune cell activity.** (**Top**) The isolation of autologous T cells enables *ex vivo* reprogramming and expansion of antitumor T cells for adoptive cell therapy. The magnitude of the immune response (i.e. output) can be titrated by varying the location, duration, and intensity of the remote-controlled trigger input. (**Bottom**) Remote triggers initiate the activation of a synthetic gene circuit to modulate programmed functions.

Advances in synthetic biology are enabling powerful new approaches to modulate immunity to increase the precision of synthetic immune cell therapies by remote and noninvasive control. Living cells use diverse biological mechanisms to sense, process, and respond to their dynamic surroundings. These biological components - such as ligands, receptors, and signaling pathways - can be rewired into complex biocircuitry to sense-and-respond to multiple inputs, including externally applied stimuli, based on logical computation 7, 8. Therefore, immune cell therapies that are genetically engineered with remote controlled circuits allow for the noninvasive and site-directed activation of therapeutic programs capable of tuning the potency, specificity, and safety of engineered immune responses. This review highlights emerging strategies for engineered immune cell therapies that are controlled by exogenous inputs - from light, heat, and biophysical cues - and endogenous inputs local to the disease microenvironment, such as dysregulated protease activity, for synthetic immunity. The focus will be on adoptive T cell therapies, but the technologies described have broad applications for control of macrophages, NK cells and other immune cells to potentially increase the precision and safety of therapeutic interventions against different diseases (**Figure 1**).

## Synthetic Gene Switches for Remote Control of Mammalian Cells

**Figure 2:**
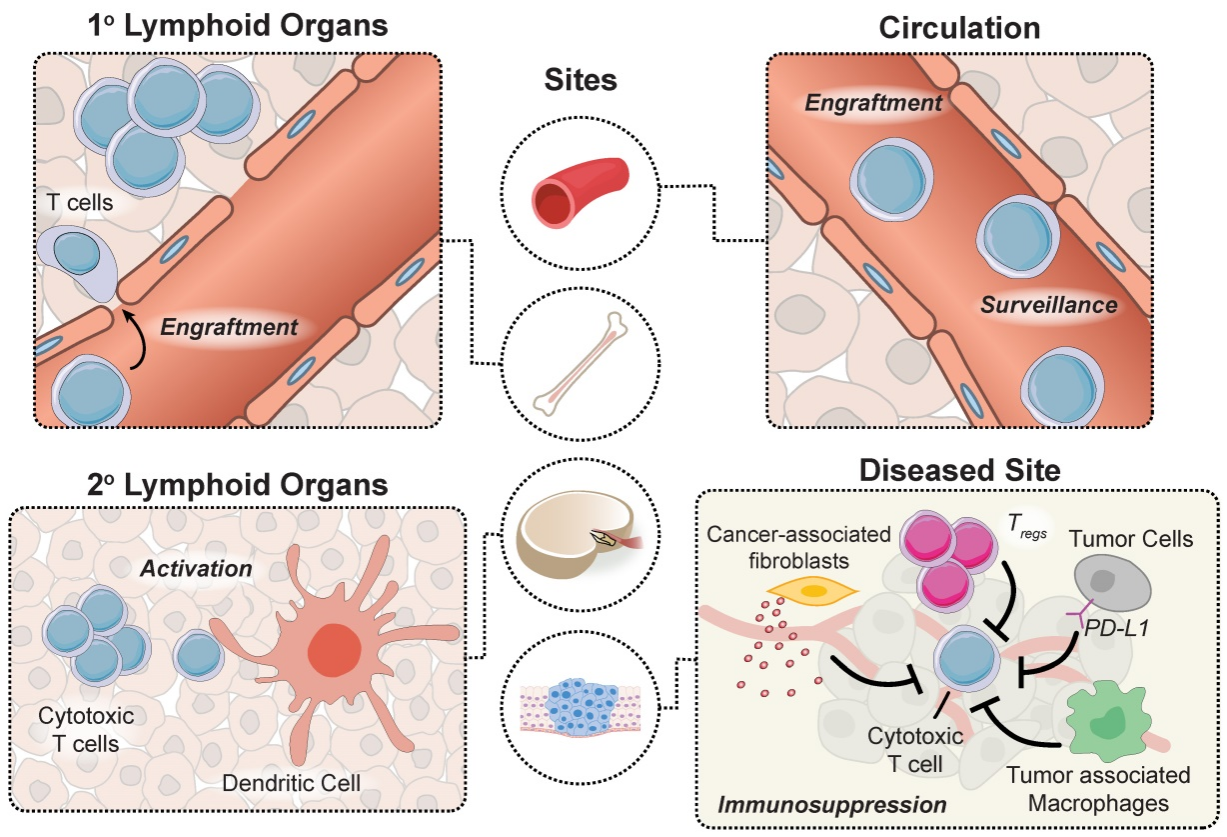
** An effective antitumor response requires unique interactions of T cells in different immunological sites.** Produced in the bone marrow, T cells move to the thymus where they mature and differentiate into various subtypes before trafficking to secondary lymphoid organs for priming by DCs. T cells subsequently enter circulation and transport to diseased sites expressing cognate antigens, where they must overcome immunosuppressive signals to effectively clear malignant cells.

Adoptively transferred T cells interact with cellular and microenvironmental signals at distinct anatomical sites throughout the body during a successful antitumor response. Prior to autologous T cell transfer, patients undergo lymphodepletion to remove recipient T regulatory cells in the periphery and improve engraftment of adoptively transferred cells in the marrow 9. To proliferate and prolong the persistence of circulating T cells, patients receive infusions of cytokines (e.g. IL-2) and/or professional antigen presenting cells (APCs) such as dendritic cells (DCs) loaded with tumor antigens that provide positive stimulatory signals. APC activation occurs within tumors in tertiary lymphoid centers, as well as in secondary lymphoid organs such as tumor draining lymph nodes. Neoantigens that are released by dying tumor cells are loaded by APCs to expand the endogenous T cell response via classical binding of T cell receptors (TCRs) to peptide-MHC complexes and engagement of CD28 receptor to B7 (CD80/86) molecules expressed on the surface of APCs that trigger costimulatory signals 10. Although CARs are not restricted to antigen recognition on peptide-MHC complexes, strategies developed to prime CAR T cells *in vivo* demonstrated enhanced antitumor function 11-13. Once they reach tumor sites, engineered T cells experience an immunosuppressive tumor microenvironment, including tumor associated macrophages, T regs and myeloid derived suppressor cells (MDSCs), and immune checkpoint inhibition through the PD-1 and CTLA-4 pathways that represent significant barriers to effective anti-tumor responses. These major steps along the tumor immunity cycle occur at distinct anatomical sites and therefore, represent unique opportunities for site-specific remote modulation of immune cell therapies (**Figure 2**). The following sections will review different approaches for remote control of cell activity and how these strategies synergize with engineered T cell therapies to augment synthetic immunity.

### Small molecule-based triggers

The ability to remotely control the activity of engineered immune cells after adoptive transfer has numerous applications 14-16. During tissue repair, for instance, distinct patterns of cytokine and transcription factor expression are associated with T reg modulation of neutrophil clearance 17, macrophage polarization 18, and regulation of helper T cells 19. Epigenetic landscapes and chromatin structures have also been linked to T cell exhaustion, memory, and effector phenotypes 16. Of note, others have described a stem-like CD8 T cell population that is characterized by low checkpoint molecule expression, high expression of costimulatory molecule CD28 and high expression of transcription factor TCF1 20-22. Within tumors, these stem-like CD8 T cells support the antitumor T cell response by maintaining the ability to proliferate while simultaneously giving rise to effector cells 22. These examples highlight opportunities to modulate immunity by reprogramming T cell function. However, it is important to develop strategies that enable careful management and dynamic control of engineered cell programs. This section will explore the use of small molecules as triggers for control of synthetic immune responses 23, 24.

**Figure 3:**
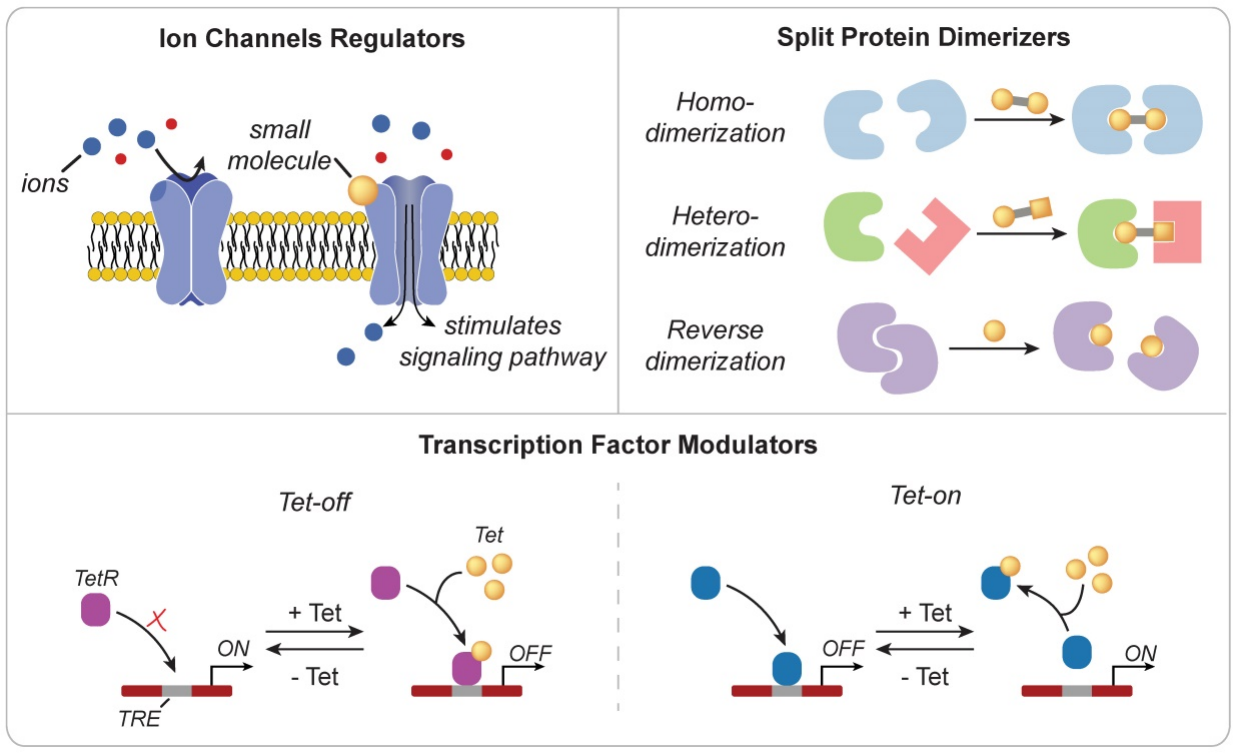
** Mechanisms of action for small molecule triggers.** (**Top Left**) Ion channel activity may be gated by small molecules to regulate signaling pathways (**Top Right**) Small molecules can also be engineered as dimerizers to control the functional state of proteins (**Bottom**) Small molecules can effectively sequester transcription factors through a steric hindrance mechanism to regulate transcriptional activity.

Small molecules are low molecular weight organic compounds that can regulate biological processes through a variety of mechanisms (**Figure 3**). Those engineered to modulate the activity of surface proteins, such as ion channels or TCRs, have been used to control downstream signaling pathways 25, 26. For example, Wong and Wong engineered ZAP70, a cytoplasmic tyrosine kinase that is naturally recruited to the intracellular domain of TCRs following stimulation, to be sensitive to small molecule regulation 27. Upon administration of the small molecule 4-hydroxy-tamoxifen (4-OHT) or 3-MB-PP1, the synthetic ZAP70 protein was either recruited to the TCR to initiate downstream signaling or inhibited from transmitting signals in a dose dependent manner. Small molecules can also be used to dimerize protein fragments that are otherwise nonfunctional. One of the most widely used dimerizer pairs is the FK506 binding proteins (FKBP) and FKBP rapamycin binding (FRB) domain of mTOR where the small molecule rapamycin induces dimerization of FKBP and FRB 28. These dimerizers have been used to functionalize split Cre recombinase 29, initiate signaling cascade to induce gene activation 30, and promote oligomerization of multi-ordered proteins 31. Zetsche and colleagues also designed a split Cas protein for conditional control of genome editing and transcriptional modulation with rapamycin 32. Leveraging the versatility of CRISPR-Cas tools is particularly useful in applications that require control over multiple genes (e.g. cytokines and checkpoint molecules) or manipulation of epigenetic landscapes (e.g. T cell exhaustion or effector phenotypes) to alter immune cell function 33, 34. However, at high doses, the side effects of small molecules, such as the immunosuppressive activity of rapamycin 35, may limit potential use for immune cell therapies. As such, alternate or synthetic small molecules are actively being explored 36-38.

Developed in 1992 and inspired by the Tn*10*-encoded tetracycline-resistant operon of *E. coli* 39, the Tet-On/Tet-Off system provides another approach for controlling transcriptional activity. In the original Tet-On system, administration of the small molecule tetracycline inhibits binding of the tetracycline repressor protein (*tet*R) to DNA-binding motifs known as tetracycline operators (*tet*O) found within tetracycline-responsive promoters. This interaction relieves the suppressive activity of TetR and allows for transgene expression. Tet-regulated control of IL-12 expression in cancer-specific T cells enhanced antitumor immunity in a murine model of melanoma without the side effects associated with systemic administration or expression by a constitutive promoter 40. Several groups have also demonstrated that the conditional expression of CARs using the Tet-On system mitigates adverse effects associated with constitutive expression 41, 42, as well as T cell exhaustion associated with tonic signaling via the chimeric antigen receptor 43. To improve *in vivo* efficacy, transcriptional regulators of the Tet system (e.g. rtTA) have been optimized to increase sensitivity to tetracycline, reducing the potential for drug-associated side effects and expanding control to tissues characterized by poor drug delivery profiles 44. Direct interaction with genomic DNA using small molecules also enables control of transcriptional activity 45-48. Distamycin A and hedamycin, which are minor groove-binding intercalating ligands, can non-specifically bind to TATA boxes and G/C rich regions respectively to downregulate transcription of downstream genes 46. On the other hand, heterocycles comprised of imidazole, pyyrole, and hydroxypyyrole, may function as transcription factors that selectively bind to target DNA sequences with affinities and specificities comparable to DNA-binding proteins 47.

As engineered T cell therapies become more prevalent across the clinic, associated life-threatening toxicities, such as cytokine release syndrome (CRS), neurologic toxicity, and “on-target/off-tumor” recognition 6, 49, underscore the need to develop strategies to rapidly attenuate responses should the need arise. In the event of CRS, for instance, symptoms can arise within minutes after infusion. In one example, a cancer patient treated with anti-HER2/neu CAR T cells experienced symptoms of respiratory distress as early as 15 minutes after infusion, and quickly developed severe CRS that led to multi-organ failure and death 50. The rapid timescales with which these symptoms can manifest highlight the need to integrate tight control over when engineered cells are active. One approach is to engineer a permanent OFF, or “kill”, switch within CAR T cells using a 4-OHT inducible Cre recombinase (MerCreMer) 51. In this system, the Cre recombinase is fused to two mouse estrogen receptor (Mer) subunits. In the absence of 4-OHT, the binding of the Mer subunits to HSP90 sequesters the Cre recombinase in the cytosol. Upon administration of the small molecule trigger 4-OHT, HSP90 is displaced and Cre translocates into the nucleus where it acts on two genetic *loxP* sites to permanently excise, invert, or translocate a genetic element 52, 53. Alternatively, CAR expression can be conditionally activated using small molecules. Such a switch allows for titratable control of CAR expression, and complete inactivation with the removal of the drug stimulus 54.

The diversity of distinct small molecule compounds and the ease of their delivery by injection, ingestion 55, or inhalation 56, highlight the potential of using chemical triggers to control immune cell function. Recent demonstrations include the activation of TLR signaling pathways 57, modulation of CAR T cell function 54, 58, and reversal of T cell exhaustion 59. A drawback of small-molecule control is that they are administered systemically, which limits applications where local control of immune responses is critical, especially for mitigating off-target toxicities that have hindered the success of many immunotherapies 60, 61. Delivery can also be challenging to target to disease sites, such as to malignancies in the brain, or to tumors with significant extracellular matrix (e.g., pancreatic adenocarcinomas) 62 due to mass transport barriers. The next sections will discuss light- and heat-based systems that can be used to locally target engineered cells to improve spatial control of immunity.

### Light-based gene switches

The use of light as a remote trigger provides local control of engineered cell activity. Already, examples of light-triggered systems for macrophage 63, T cell 63-66, and DC activation 63, as well as CAR expression 67 demonstrate the potential for applications in synthetic immunity. For example, He *et al*. showed reversible, light-dependent control of Ca^2+^ influx in multiple immune cells 63. T cell stimulation through the TCR in the presence of costimulatory signals results in an intracellular calcium increase and subsequent activation of the nuclear factor of activated T cells (NFAT) signaling pathway. The NFAT family of transcription factors are calcium-dependent regulators of T cell activation, differentiation, and development 68. Engineered control of NFAT regulation is a promising approach for immunomodulation of T cells. Optical regulation of Ca^2+^ signaling coupled with the addition of phorbol 12-myristate 13-acetate (PMA) to mimic costimulatory signals, led to T cell activation upon light stimulation as evidenced by increases in IL-2 and IFN-γ production 63. Alternatively, the NFAT pathway can be used to regulate transgene expression using NFAT promoters. Control of immunomodulatory genes (e.g., IL-2, IL-15, TNF-α) using these promoters allows for light-mediated control of T cell activity for enhanced antitumor efficacy 66. In another approach, Allen and colleagues utilized photosensitive dimerizers to confine CAR expression to cells illuminated by light 67, highlighting a strategy to potentially mitigate off-tumor activation of CAR-mediated toxicity. The creation of light-based switches such as these is made possible by photosensory proteins or domains, such as melanopsin and CRY2, that undergo conformational changes upon light stimulation 69, 70. The focus of this section is on the application of these light responsive units for the remote modulation of immune cells.

Opsins, light-gated G-protein coupled receptors naturally found in animals, can be repurposed to initiate genetic programs upon exposure to light. Melanopsin, for instance, has been integrated into primary human T cells to induce localized production of cytokines IL-2, IL-15, and TNF-α to promote T cell mediated killing of solid tumors in response to blue light (ʎ = ~450-480 nm) 66. Rhodopsin, on the other hand, has been reengineered for optical control of chemokine signaling to direct T cells to recruit T cells to tumor sites upon illumination with green light (ʎ = ~480-500 nm) 71. However, the penetration depth of blue light is limited by tissue scattering and absorption; as such, groups have developed red light enabled genetic toggles to achieve deeper penetration. Similar to melanopsin, the red-light sensitive channel BphG1-Sir1143 (BphS) has been employed to produce cyclic di-GMP for downstream signaling 72. Nonetheless, penetration of light from lasers or intense pulsed light (IPL) devices remain limited to superficial targets located within the dermis, a penetration depth of only several millimeters for red light 73. To improve local access, light emitting diodes (LEDs) can be implanted directly at the desired site of control. Folcher *et al.* generated a system in which a subcutaneously implanted, wireless light was powered through a mind-controlled induction coil to activate transgene expression of mammalian cells in mice 74. He *et al*. boosted the penetration depth of light activated systems using lanthanide-doped upconverting nanoparticles, which convert a penetrative wavelength (i.e. near infrared or NIR) to a stimulating wavelength (i.e. blue light), to initiate DC mediated activation of CD8+ T cells 63. These nanomaterials act as *in situ* nano-illuminators to locally activate light-responsive engineered immune cells.

The use of photocaged molecules provides another avenue for light-based control systems. Rather than inducing a chemical signaling pathway, a molecule capable of altering gene expression is inactivated by a light-cleavable moiety that is removed upon light irradiation 75. An early example of photocaged control was reported by Cruz *et al*. where a non-agonist, photocaged estradiol molecule was synthesized and delivered to HEK293 cells. Upon UV light exposure, the estradiol molecule became functionalized to initiate estrogen receptor (ER)-mediated transcription 76. Light-based control was later expanded to include IPTG for the *lac* operon, doxycycline for the Tet-on/off systems, and rapamycin for FKBP/FRB dimerization under blue light 75. Of note, this technique was used to modulate immunity via TLR signaling. Much like adjuvants promote DC activation and induce IFN-γ to enhance vaccine efficacy and long-term immunity 77, DC activation by TLR agonists induces the expression of costimulatory signals and cytokine expression patterns to promote the functional differentiation of CD8+ T cells 78. Due to the ability of DCs to shape immunity, a photocaged TLR 2/6 agonist was designed to bind to DCs, such that upon light stimulation, the freed agonist triggers TLR signaling to induce T cell recruitment and priming in illuminated lymph nodes 79.

Light-sensitive systems have also been designed to impart control over gene expression using photosensitive dimerizers, such as Cry2/CIB1 80, pMag/nMag 81, or GAVP 82. Similar to small molecule dimerizers used to induce assembly of a functional CAR 54, photosensitive proteins could be used to regulate CAR-mediated killing with the added benefit of site directed control 83. Although photosensitive dimerizers have yet to be integrated for the regulation of CARs, such a design strategy would enable reversible control with the potential to mitigate off-tumor toxicity and T cell exhaustion due to chronic signaling. The integration of multiple circuits activated by distinct wavelengths, however, may be difficult to achieve due to unintended activation caused by spectral overlap. For this reason, Müller *et al.* developed a model in which PhyB-PIF6, LOVpep-PDZ, and UVR8-COP1 show orthogonal dimerization upon red, blue, and UV-B controlled stimulation, suggesting that a single system can be developed to respond differentially to three distinct light stimuli 84.

Additionally, the system termed light-inducible transcription using engineered zinc finger proteins (LITEZ) was designed to regulate genomic transcriptional activation. LITEZ combines blue-light induced protein dimerization to localize a VP16 transcriptional activator to zinc finger proteins bound upstream of a gene of interest, and thus allow for remote activation of endogenous genes 85. A similar system was integrated with a dCas9 protein in which a Cry2/CIB1 dimerization event localizes a strong transcriptional activator (VP64) to a dCas9 to induce transcriptional control over genes downstream of dCas9 binding 86. Moreover, by using a catalytically active split Cas9 system, Zhou *et al.* implemented similar light induced dimerization peptides to regulate gene editing by remote blue light control 87. Several groups have implemented Cas-mediated multiplex gene regulation to induce or discover therapeutic cell phenotypes, including the induction of cell differentiation 88, 89 and the use of genome wide gain-of-function or loss-of-function screens to identify new targets to enhance the antitumor activity of cytotoxic T cells 90, 91. As such, optogenetic control of CRISPR-Cas9 tools offer to provide a multiplexed approach to remotely modulate immune cell phenotypes directly *in vivo*.

Light-based methods increase the precision of immune control by allowing key sites in the body to be targeted. Similar to chemically inducible systems, light-activatable constructs can be controlled with temporal accuracy, but more importantly, they offer the ability to rapidly modulate cells with remote spatial control (**Table 1**). With this, cell activity such as cytokine secretion 66, CAR expression 83, and chemotactic T cell migration 71 can be confined to anatomical sites that require therapeutic intervention. However, to expand the broad applications of remote-controlled systems, strategies that improve penetration depth of the light are necessary to increase access to tissue. Ongoing development of gene switches activated by NIR light, whose characteristic wavelength (ʎ = 600-1000 nm) nearly doubles effective penetration up to a few millimeters (~5 mm 83, 92), is enabling the use of photoinducible systems at increased depths. These include the *de novo* design of NIR responsive proteins to expand the library of light responsive elements 93 and the use of upconverting nanoparticles, which enhance the penetration depth using NIR light to impart control over the wide array of existing photosensitive proteins 63, 92, 93. And yet, many target tissues remain inaccessible by light. This warrants the need for strategies that can impart local control of engineered cells, even at sites difficult to reach by noninvasive means, such as intracranial malignancies and deep lymph nodes.

### Heat as a remote trigger

**Figure 4:**
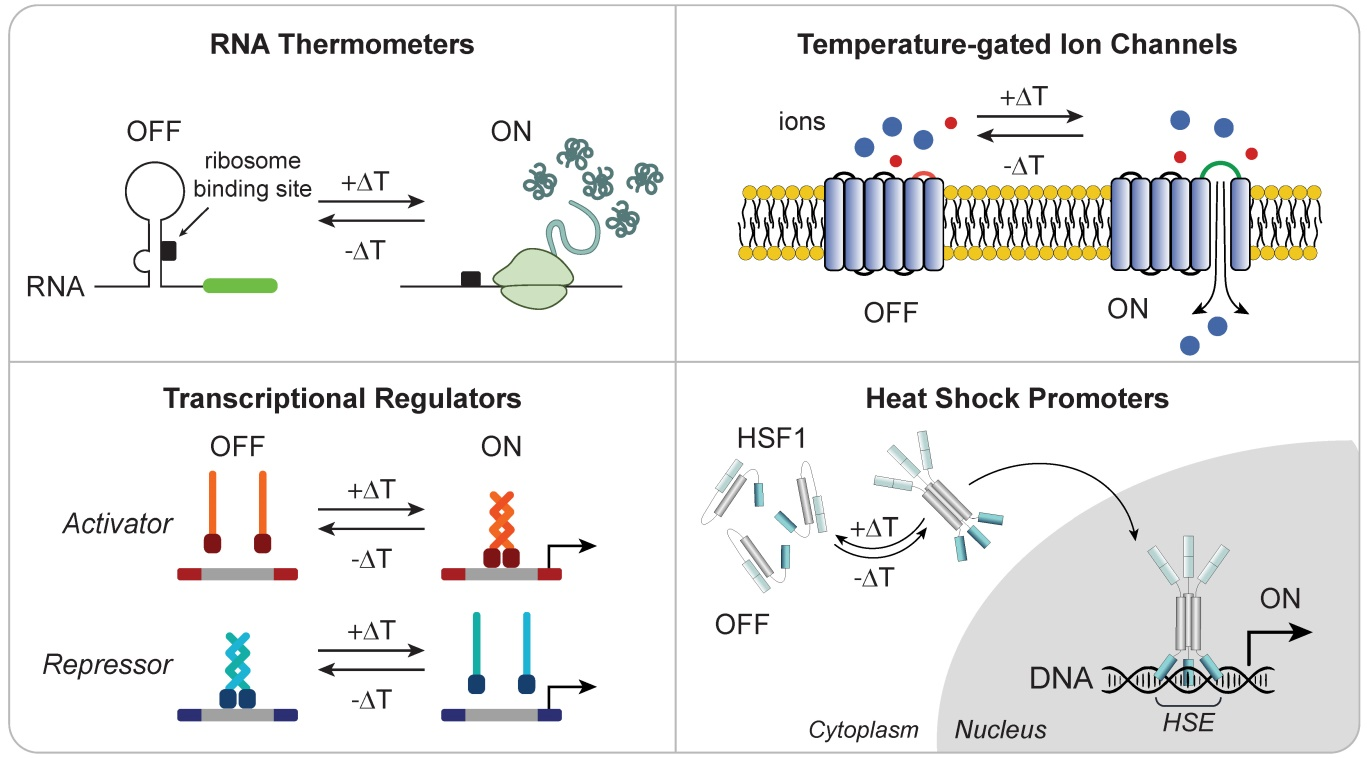
** Heat responsive molecular components for thermal regulation of engineered gene expression.** (**Top Left**) RNA thermometers protect ribosome binding site until heat-triggered conformational change allow translation of mRNA. (**Top Right**) Temperature-gated ion channels open in response to thermal change. (**Bottom Left**) Transcriptional regulators with thermal sensitivity allow for DNA activation or repression upon heat stimulation. (**Bottom Right**) Heat shock induces trimerization of HSF1 monomer allowing for its translocation into the nucleus where they bind to HSEs to initiate transcription of heat shock proteins.

Localized thermal control of tissue temperature has potential as a noninvasive trigger for immune cell therapies. Hyperthermia is used in the clinic for applications such as increasing perfusion of drug delivery 94, sensitization to radio- and chemotherapy 95-98, and thermal ablation of tumors 99. Pulses of heat can be delivered noninvasively and with millimeter precision to deep anatomical sites by several platforms, such as high-intensity focused ultrasound (HIFU) 100 or magnetic particles in alternating magnetic fields 101. Exposure to mild hyperthermia (~39-42°C) induces the heat shock response (HSR), a highly conserved molecular response to cellular stress that leads to transient expression of cytoprotective genes known as heat shock proteins (HSPs). Transcriptional induction of HSPs is primarily mediated by the evolutionarily conserved transcription factor, heat shock factor 1 (HSF1); upon cellular stress, latent HSF1 monomers are released from an inhibitory multichaperone complex 102, enabling it to form homotrimers with high affinity to DNA. These complexes then translocate to the nucleus and bind to DNA motifs called heat shock elements (HSEs) within promoters of HSPs to drive transcription to levels comparable to the strongest viral promoters known 103. Recent control methods based on heat-induction to modulate gene expression 104-106 leverage the use of both endogenous and synthetic components that respond to changes in temperature, including RNA thermometers 104, temperature-gated ion channels 107-109 and transcriptional regulators 105, as well as highly-inducible heat shock promoters 110-119 (**Figure 4**).

While eukaryotes and prokaryotes have evolved highly homologous HSRs, the basic molecular mechanisms that underlie a cell's thermosensory capabilities differs among organisms. Microorganisms rely on the heat shock response for sensing and survival, as they lack internal mechanisms for regulating cellular temperature. For example, pathogenic bacteria rely on temperature to sense invasion of the mammalian host to trigger the expression of virulence genes 104. These cells exploit temperature-sensing RNA molecules, called “RNA thermometers”, in complex secondary structures to shield the ribosome binding site (RBS) until a temperature-dependent conformational change exposes the site for translation. While RNA thermometers are primarily found in prokaryotic systems, early evidence suggests they may also be integral mediators of mammalian HSR 120. Due to their tunable properties, such as temperature thresholds and fold-inductions 121, RNA sensors are attractive candidates for building synthetic thermal switches 122, despite that the prokaryotic origin of these bacterial circuit components could potentially lead to immunogenicity in mammalian systems.

Exploiting the robust mechanism by which trimerized HSF1 binds to HSEs to drive the expression of target chaperone proteins, another class of thermal gene switches use heat shock promoters to drive transgene expression. Among these, promoters of the HSP70 family have been widely explored for heat-inducible transgene expression due to high inducibility 110-116. Early work successfully employed the HSP70B promoter to selectively trigger expression of reporter and immunostimulatory genes both *in vitro* and *in vivo* in response to exogenous pulses of heat. Huang *et al*. demonstrated up to a 1,000-fold induction over background of the reporter GFP gene when gene expression was controlled by the HSP70B promoter 112. These constructs demonstrate a dose-dependent reversible expression of transgenes, providing tunability that can be modulated by the intensity and duration of thermal treatment 110, 112. Using these constructs, site-directed control of transgene expression, including the proinflammatory cytokine IL-12, have been achieved directly *in vivo* by multiple heating modalities such as MRI-guided focused ultrasound and irradiation with NIR light or magnetic fields in combination with nanoparticles 112-116, 123. This approach provides a strategy for safely controlling potent genes that are typically toxic when administered systemically. For example, intratumoral injections of adenovirus carrying the genetic circuit for HSP70B-driven expression of the immunostimulatory cytokine interleukin-12 (IL-12) cause significant delay in tumor growth only in heated lesions in a murine model of melanoma 112, while systemic administration of recombinant IL-12 is otherwise associated with severe toxicity 124.

HSP promoters respond broadly to different types of cellular stress including oxidative stress, radiation, and heavy metals 119, 125. To minimize transcriptional activation by non-thermal inputs, several groups have explored the use of the HSPA6 (HSP70B') promoter and fully synthetic constructs consisting of arrayed HSEs 117-119. The HSPA6 promoter is closely related to the HSP70 (HSPA1A) promoter, but is only found in higher mammals, including humans. A 3 kb HSPA6-derived thermal switch achieves improved fold-activation in human keratinocytes when compared to one of HSP70 origin 119. Truncation analysis of the wild-type promoter sequence identified constructs characterized by improved basal activity and fold-induction 117, 119. The reduction of non-HSE regulatory regions, such as hypoxia-response elements (HREs), alters the response profile to both thermal and non-thermal cues and enables the development of thermal switches with improved fold-activation, reduced responses to orthogonal cell stressors, and negligible basal activity 126. In addition to offering enhanced thermal control, the smaller DNA footprint of these synthetic constructs makes it much easier to incorporate with gene therapies and *in vivo* applications.

Several studies have integrated thermal control with engineered cell therapies. Miller *et al*. demonstrated the use of thermal gene switches for single-gene control in human T cells 117. To achieve broad control over cell phenotype, Gamboa *et al*. integrated an HSPA6-derived thermal switch with dCas9 constructs, providing the ability to turn on as well as suppress multiple target genes (e.g. cytokines, immune checkpoints) by introducing their cognate guide RNAs (sgRNAs) 123. dCas9 expression - when placed under control of thermal switches - was modulated by thermal triggers as few as 15 minutes in duration. Using dCas9 modified with transcriptional activators, heat activated transcription of multiple target genes was equivalent to levels that can be achieved using a strong viral promoter. Additionally, leveraging spatial targeting by laser heating, heat-triggered control of engineered T cells as well as dCas9-mediated target gene modulation was achieved *in vivo* 117, 123.

Together, these works represent a sampling of how heat can be used for remote control of gene expression. By using heat, local modulation of transcription can be achieved noninvasively even in deep tissue, including intracranial malignancies 127. The potential applications of this approach are complementary to existing small-molecule and optogenetic tools (**Table 1**). In comparison to chemical or light-inducible constructs, which use split proteins to achieve rapid formation and dissociation of functional complexes 128, transcriptional modulation using thermal switches is delayed relative to the input heat pulse since proteins must be transcribed and translated prior to being functional. This also means that OFF kinetics are governed by protein degradation rates, and therefore activity can be maintained for several days by delivering short pulses of heat on the order of minutes. The lack of need for continuous stimulation may facilitate the design of preclinical animal studies, and aid future translation. However, because thermal switches may rely on components of the endogenous heat shock response, characteristics of heat pulses (e.g. temperature, duration, frequency) must be carefully considered. Therefore, further characterization of the thermotolerant state and cellular responses to heat shock and may be needed to optimize thermal inputs.

### Biophysical Cues

Biophysical cues such as mechanical and magnetic triggers offer orthogonal approaches to small molecule, light, and heat triggers for controlling gene expression 129-132 (**Table 1**). Mechanically induced circuits continue to emerge as advancements in cellular imaging and force-sensing tools provide a new understanding of cell surface receptors and their responses to mechanical forces to trigger intracellular signaling and response 130. Mechanically sensitive receptors translate a mechanical force into a chemical cascade. First discovered in neural cells in 2010, the surface Piezo1 receptor triggers downstream signaling after mechanical stimulation 129, 133. Pan *et al*. demonstrated that T cells engineered with a Piezo1 receptor can express CARs following mechanical stimulation by focused ultrasound in combination with microbubbles 129. Microbubbles amplified the mechanical forces and stimulated the Piezo1 receptor to induce a calcium influx and subsequent NFAT transcriptional pathway. In rewiring cell circuitry to respond to NFAT signaling with transgene CAR production, this work demonstrated mechanically-dependent CAR T cell killing *in vitro* 129. Other receptors, such as the Notch receptor have also been heavily utilized in mechanogenetics. Notch receptors were discovered in the late 1990s for their importance in T cell development and induce downstream signaling upon ligand engagement and force stimulation 134. Leveraging Notch receptors, Seo *et al.* induced transgene reporter expression following a mechanical trigger 130. Notch receptor-homing magnetoplasmonic nanoparticles (MPNs) bind to the engineered cell surface receptor, which is intracellularly bound to a GAL4 transcriptional activator. Upon application of a magnetic field, an external mechanical force leads to intracellular peptide cleavage, and thus release of GAL4 to allow for translocation in the nucleus and subsequent transgene activation 130.

Besides the use of a magnetic field to induce mechanical 130, 135 or thermal changes 136, the direct effect of magnetism can act as a mode of gene regulation due to the ability of magnetic fields to pass freely through organic tissue 137. Cell receptors comprised of iron-sulfur cluster assembly protein 1 (Isca1) are iron containing magnetoreceptors that can be found in the plasma membrane. Upon activation by a remote magnetic field, Isca1 polarizes the membrane and generates a calcium influx to regulate gene expression 138. Other groups have coupled a baculoviral vector (BV) derived from a cylindrical insect virus with magnetic iron oxide nanoparticles to generate magnetic gene regulation 132. Upon targeted application of a ~1.5T magnetic field, systemically delivered MNP-BV are endocytosed by cells and the packaged DNA is delivered. Zhu *et al.* showed tissue specific transgene expression *in vivo* and utilized these particles to deliver CRISPR-Cas9 system 132 which can be used to edit or regulate genomic DNA thus providing remote gene regulation.

### Autonomous Systems

**Figure 5:**
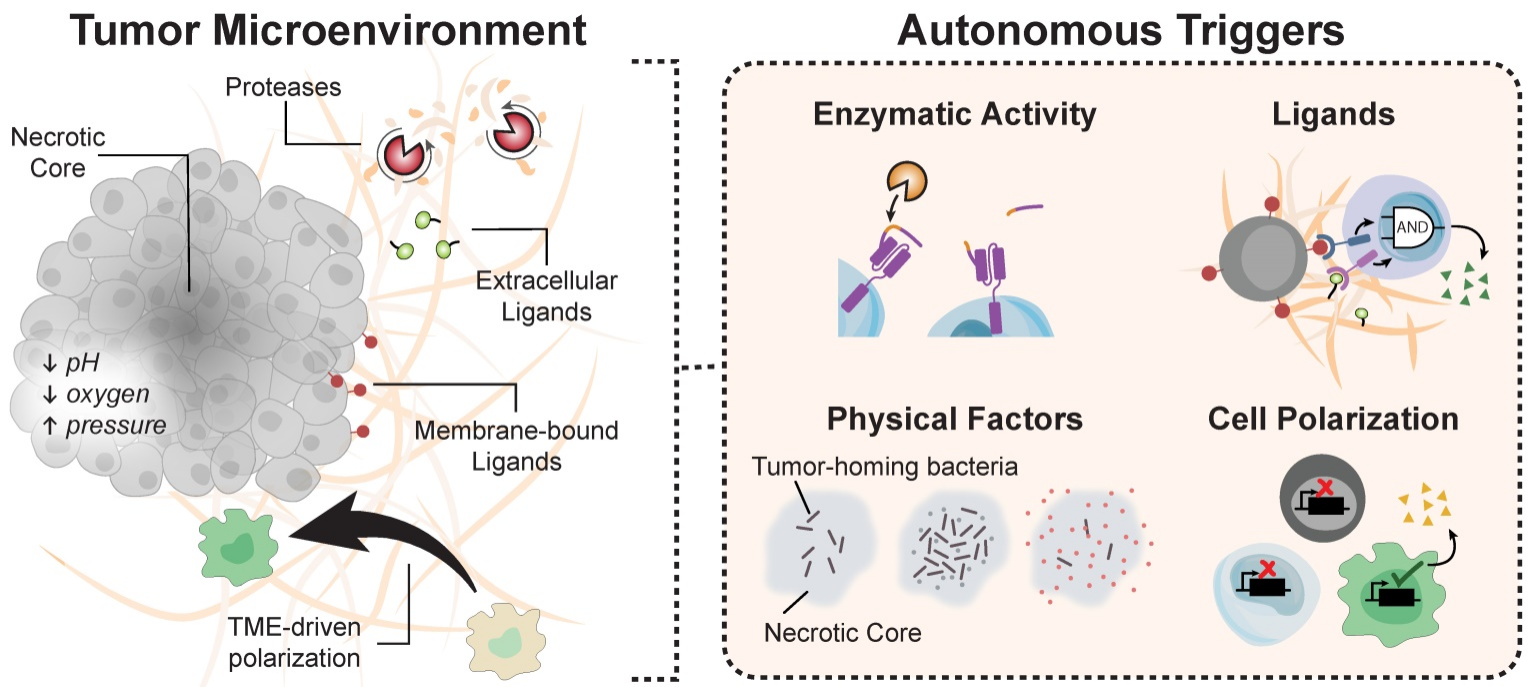
** Environmental cues for autonomous systems.** The tumor microenvironment harbors a myriad of biological molecules that engineered circuits can exploit for autonomous activation.

T cells sense-and-respond to dynamic environmental cues to traffic throughout the body, infiltrate tissues, and survey the microenvironment. The design of biocircuitry responsive to autonomous triggers allows engineered cells to be rationally controlled without the need for external triggers. Examples of environmental cues include a unique combination of surface or matrix-bound ligands, dysregulated enzymatic activity such as proteases, and biophysical factors (e.g., low pH, or hypoxia) that are characteristic of tumors compared to healthy tissue (**Figure 5**). The emerging autonomous systems described in this section offer novel strategies to sense key indicators of disease, complementing remotely triggered systems. To provide the ability to sense-and-respond to different target ligands, for instance, the Notch protein, a highly conserved transmembrane receptor which releases an intracellular transcriptional domain upon extracellular engagement, was reengineered as a synthetic Notch (synNotch) to drive signaling upon recognition of target extracellular ligands 139. Similarly, CARs, which combine the binding affinity of an extracellular scFv with intracellular signaling domains for activation, allow T cell cytotoxicity to be redirected toward target antigens without the requirement for MHC-restricted antigens 140.

During T cell activation, the surface redox activity increases, a property which can be used as an autonomous chemical trigger to control the local release of potent biologics. Tang *et al*. designed nanogels (NGs) comprised of the IL-15 superagonist crosslinked by reversible thiol bonds to form protein “backpacks” on the surface of T cells. Upon antigen recognition and an increase in cell-surface reduction activity due to TCR activation, IL-15 superagonist is released locally into the microenvironment to concentrations eight times greater than are safely achievable by systemic administration, enhancing T cell proliferation and increasing tumor cell cytotoxicity 141. To increase the precision of tumor cell recognition, strategies that incorporate two-input autonomous triggers for logic-gated sensing have been developed. The scarcity and heterogeneity of tumor associated antigens (TAAs) has severely limited the ability to treat solid cancers with engineered T cells 142. Many TAAs share expression with healthy tissue (e.g. HER2, EGFR), leading to on-target/off tumor toxicities 143. Furthermore, targeting specific antigens can render T cell therapies ineffective against tumors that lack expression of the target antigen due to antigen loss or heterogeneity 144. Strategies that incorporated logic-gated sensing have demonstrated therapeutic efficacy in preclinical models. These include OR-gated CARs that mitigate antigen escape by targeting both HER2 and IL13Rα2 in a mouse model of glioblastoma 145. Similarly, AND-gated CARs have been designed to increase the specificity towards tumor cells in a preclinical model of human prostate cancer by requiring the presence of both PSMA and PSCA antigens for T cell activation 146. To further minimize “on-target, off-tumor” toxicities, Federov *et al*. designed a NOT-gate circuit with competing CARs to inhibit cytotoxicity when a healthy antigen is recognized 147.

Cell polarization induced by the tumor microenvironment can also serve as an autonomous trigger. Aalipour and co-authors reported the concept of cell-based “immunodiagnostics” by exploiting M2 polarization of macrophages within the TME, which upregulated the Arginase 1 promoter by as much as 200 fold *in vivo*, as a selective trigger to release a secreted biomarker in adoptively transferred macrophages as a cell-based diagnostic 148. Without TME cues, these engineered macrophages do not polarize, thereby ensuring detection signals are tumor-specific. Other TME characteristics, including low oxygen and acidic conditions (**Figure 5**), are also promising targets for autonomous control of immunity 149. Hypoxic and acidic conditions activate promoters upstream of stress proteins, such as the *HIF1α* 150, 151 and *Gas* 152, which can be reengineered as synthetic gene switches. While the availability of pH dependent promoters sensitive to the TME are currently limited to highly acidic conditions (pH ~2-3) 152, 153, pH-responsive nanoparticles have been engineered to change their soluble/insoluble state in response to changes in external pH levels characteristic of the TME (i.e. pH ~6) thus releasing cargo locally into the extracellular fluid 154, 155. Moreover, gold nanoparticles capped with carboxymethyl chitosan can release cationic molecules in response to acidic pH 156. While the use of hypoxia- and pH-inducible systems has been heavily explored in nanomedicine 157, 158, these autonomous cues have yet to be implemented for direct control of immune cell activity to the knowledge of the authors. Such approaches remain promising, as they may provide ways to overcome the immunosuppressive milieu of the tumor microenvironment 159, 160.

Ligands that are aberrantly overexpressed within the TME can also direct autonomous activation. TGF-β, for instance, promotes cancer progression by inhibiting cytotoxic T cell activity 161, yet Chang *et al.* designed CAR T cells capable of converting the immunosuppressive TGF-β signal into a potent T cell stimulant 140. Upon stimulation by soluble TGF-β, the production of proinflammatory cytokines TNF-α, IL-2, and IFN-γ by αTGF-β CAR T cells led to the expansion - rather than the suppression - of primary human T cells 140. In addition to the need for developing strategies to overcome T cell immunosuppression, the scarcity of tumor-associated antigens (TAAs) has led to adverse effects that severely limit the clinical translation of promising T cell therapies 142**.** Many validated TAAs share expression with healthy tissue (e.g., HER2, EGFR), leading to potentially lethal on-target/off tumor toxicities 50, 143. To improve selectivity to tumors, Han and colleagues masked the extracellular scFv of an αHER2 CAR with a protease-cleavable domain specific for a cocktail of tumor-associated proteases. Upon entering the tumor microenvironment, these proteases cleave off the peptide mask and expose the antigen binding domain of the chimeric receptor 61, ultimately reducing the potential for off-target engagement of the CAR T cell.

Another strategy for improving the tumor specificity of engineered T cells is the integration of Boolean logic with remote triggers, such as light-, heat-, or small molecule stimuli, to mitigate off-target gene activation 111, 162-164. For example, an AND-gate system requiring thermal and chemical inputs functions by initiating transcription of a rapamycin dependent heterodimer pair through a heat shock promoter 162. This dual-gated system is characterized by minimal background activation and sustained transgene expression that remains silent upon stimulation by a single stimulus. Similar systems were developed to incorporate light and chemical cues instead 67, 163. For example, by requiring secondary input to induce CAR expression 67, the incorporation a light-based AND-gate to an existing chemically controlled Cre system overcomes spontaneous recombinase activity characteristic of *in vivo* Cre-ERT2 applications 67, 165. Clinical applications of remote triggers require robust feedback and, together, the combinations of autonomous and remote triggers allow for the integration of diverse and precise inputs for increased control of cellular activity for synthetic immunity.

Beyond the use of engineered T cells, bacteria also have potential as autonomous therapeutics to complement cancer immunotherapies. While the antitumor activity of cytotoxic T cells can be dampened by the immunosuppressive TME, the use of bacteria to deliver potent therapeutic payloads has the potential to reduce toxicities associated with systemic administration. The use of bacteria to induce antitumor responses was documented as early as the 1890's by Dr. William Coley who observed tumor regression in a fraction of patients given intratumoral injections of heat-inactivated bacterial extracts 166. Remarkably, recent studies have shown that certain strains of bacteria preferentially colonize and grow in tumors, thriving on the nutrients of the necrotic core while masked from immune surveillance by tumor-induced suppression 167-169. This ability of bacteria to autonomously target and colonize tumor sites after intravenous or oral gavage 170 has raised the possibility of using microbes as programmable therapeutic vehicles to promote antitumor activity through local control of host immune cells. In recent studies, programming bacteria with quorum-sensing circuitry allowed bacteria to synchronize growth cycles to detect heavy metals or pathogen biomarkers 171, or upon reaching a threshold bacterial density, release a genetically encoded therapeutic cargo to kill tumor cells 172. Chowdhury *et al*. used bacterial circuits to release a nanobody that targeted CD47, an anti-phagocytic receptor that is overexpressed in several types of human cancers. The authors found that tumor-localized release of nanobody agonists of CD47 resulted in enhanced proliferation and activation of tumor-infiltrating lymphocytes, leading to durable and systemic antitumor immunity in syngeneic tumor mouse models 173.

Lastly, an emerging interface is the design of cell-free programmable biomaterials that augment synthetic immunity by targeting tumor or immune proteases as autonomous triggers. Programmable biomaterials exploit fundamental biomolecular properties, such as base-pairing specificity of nucleic acids to perform logic functions 7, 174, solve mathematical problems, and play games 175, 176. To interface with immunity, for example, Dahotre *et al*. designed a panel of DNA-barcoded tetramers capable of detecting antigen-specific T cell populations with single-cell resolution 177. Other approaches have focused on designing biomaterials that sense and respond to proteases 178, an important family of enzymes that are dysregulated across human diseases and used by both innate and adaptive immune cells as effector molecules. These include the classic granzyme family of serine proteases released by cytotoxic T cells to kill target cells. The ability to detect enzymes, such as the granzyme B protease released by cytotoxic T cells, has been shown to have diagnostic value as early urine biomarkers of transplant rejection 179-181 or as imaging biomarkers of tumor response to checkpoint blockade immunotherapies 182, 183. Recently, proteases have been designed into protein circuits in living cells 184, and as cell-free therapeutic biocircuits 185 by treating proteases as two-state biological bits based on cleavage activity (i.e., low or high cleavage velocity). In the latter study, this allowed the design of a biological analog-to-digital converter (ADC) to digitize input levels of granzyme B, and for autonomous drug delivery to selectively clear blood of bacteria 185. These emerging interfaces have the potential to lead to entirely new approaches for programmable immunity.

## Concluding Remarks

The success of CAR T cells to induce durable remission in patients with B cell malignancies is creating palpable excitement for engineering approaches to immunity. Numerous efforts are underway to address key clinical challenges that prevent broad application and effectiveness of immune cell therapies for solid tumors and diseases. Central to these opportunities is the ability to design immune cell therapies for noninvasive and remote control during all steps of an effective response within the appropriate sites within the body. Remote control of immunity may leverage external targeting with cues such as light or heat, or autonomous circuits that allow cells to sense-and-respond to local environmental signals. Emerging interfaces with seeming disparate approaches, such as bacterial targeting, may also lead to new therapeutic approaches for synthetic immunity. Looking forward, these developments will require extensive evaluation in preclinical models and careful selection of a target patient population to increase potential clinical success.

## Figures and Tables

**Table 1:**
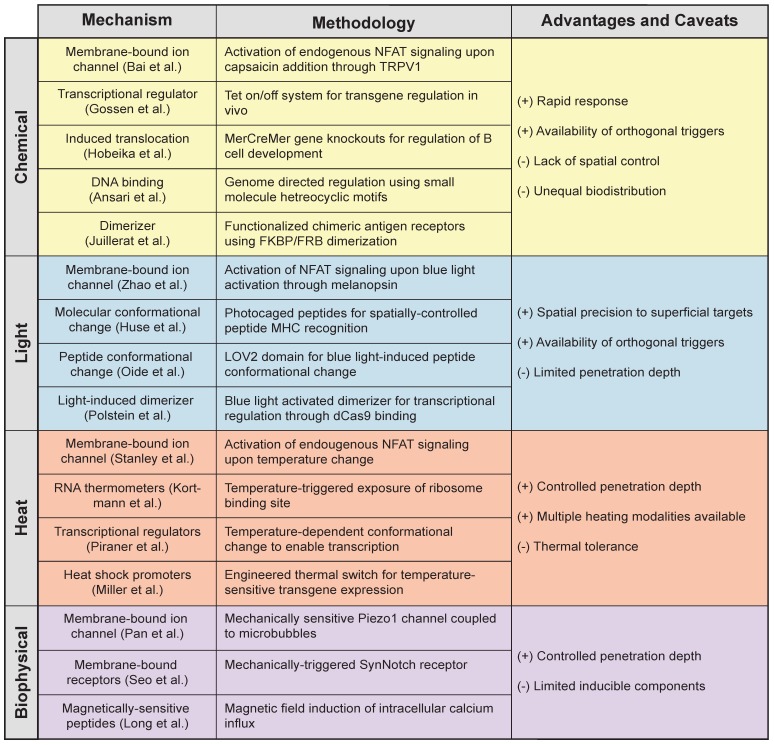
** Mechanisms of action for remote controlled triggers** 39, 47, 58, 66, 86, 104, 105, 107, 108, 117, 129, 130, 138, 186-188.

## References

[1] Bailey SR, Maus MV (2019). Gene editing for immune cell therapies. Nat Biotechnol.

[2] Xu X, Li T, Shen S, Wang J, Abdou P, Gu Z (2019). Advances in Engineering Cells for Cancer Immunotherapy. Theranostics.

[3] Porter DL, Levine BL, Kalos M, Bagg A, June CH (2011). Chimeric Antigen Receptor-Modified T Cells in Chronic Lymphoid Leukemia. N Engl J Med.

[4] Kansal R, Richardson N, Neeli I, Khawaja S, Chamberlain D, Ghani M (2019). Sustained B cell depletion by CD19-targeted CAR T cells is a highly effective treatment for murine lupus. Sci Transl Med.

[5] Skuljec J, Chmielewski M, Happle C, Habener A, Busse M, Abken H (2017). Chimeric Antigen Receptor-Redirected Regulatory T Cells Suppress Experimental Allergic Airway Inflammation, a Model of Asthma. Front Immunol.

[6] Bonifant CL, Jackson HJ, Brentjens RJ, Curran KJ (2016). Toxicity and management in CAR T-cell therapy. Mol Ther Oncolytics.

[7] Benenson Y (2012). Biomolecular computing systems: principles, progress and potential. Nat Rev Genet.

[8] Davidson EH (2010). Emerging properties of animal gene regulatory networks. Nature.

[9] Klebanoff CA, Khong HT, Antony PA, Palmer DC, Restifo NP (2005). Sinks, suppressors and antigen presenters: how lymphodepletion enhances T cell-mediated tumor immunotherapy. Trends Immunol.

[10] Xin G, Schauder DM, Jing W, Jiang A, Joshi NS, Johnson B (2017). Pathogen boosted adoptive cell transfer immunotherapy to treat solid tumors. Proc Natl Acad Sci U S A.

[11] Ma L, Dichwalkar T, Chang JYH, Cossette B, Garafola D, Zhang AQ (2019). Enhanced CAR-T cell activity against solid tumors by vaccine boosting through the chimeric receptor. Science.

[12] Wang X, Wong CW, Urak R, Mardiros A, Budde LE, Chang W-C (2015). CMVpp65 Vaccine Enhances the Antitumor Efficacy of Adoptively Transferred CD19-Redirected CMV-Specific T Cells. Clin Cancer Res.

[13] Slaney CY, von Scheidt B, Davenport AJ, Beavis PA, Westwood JA, Mardiana S (2017). Dual-specific Chimeric Antigen Receptor T Cells and an Indirect Vaccine Eradicate a Variety of Large Solid Tumors in an Immunocompetent, Self-antigen Setting. Clin Cancer Res.

[14] Li J, Tan J, Martino MM, Lui KO (2018). Regulatory T-Cells: Potential Regulator of Tissue Repair and Regeneration. Front Immunol.

[15] DuPage M, Bluestone JA (2016). Harnessing the plasticity of CD4+ T cells to treat immune-mediated disease. Nat Rev Immunol.

[16] Sen DR, Kaminski J, Barnitz RA, Kurachi M, Gerdemann U, Yates KB (2016). The epigenetic landscape of T cell exhaustion. Science.

[17] D'Alessio FR, Tsushima K, Aggarwal NR, West EE, Willett MH, Britos MF (2009). CD4+CD25+Foxp3+ Tregs resolve experimental lung injury in mice and are present in humans with acute lung injury. J Clin Invest.

[18] Weirather J, Hofmann UD, Beyersdorf N, Ramos GC, Vogel B, Frey A (2014). Foxp3+ CD4+ T cells improve healing after myocardial infarction by modulating monocyte/macrophage differentiation. Circ Res.

[19] Zheng Y, Chaudhry A, Kas A, deRoos P, Kim JM, Chu T-T (2009). Regulatory T-cell suppressor program co-opts transcription factor IRF4 to control TH2 responses. Nature.

[20] Im SJ, Hashimoto M, Gerner MY, Lee J, Kissick HT, Burger MC (2016). Defining CD8+ T cells that provide the proliferative burst after PD-1 therapy. Nature.

[21] Utzschneider DT, Charmoy M, Chennupati V, Pousse L, Ferreira DP, Calderon-Copete S (2016). T Cell Factor 1-Expressing Memory-like CD8+ T Cells Sustain the Immune Response to Chronic Viral Infections. Immunity.

[22] Jansen CS, Prokhnevska N, Master VA, Sanda MG, Carlisle JW, Bilen MA (2019). An intra-tumoral niche maintains and differentiates stem-like CD8 T cells. Nature.

[23] Gatz C (1997). Chemical Control of Gene Expression. Annu Rev Plant Physiol Plant Mol Biol.

[24] Voß S, Klewer L, Wu Y-W (2015). Chemically induced dimerization: reversible and spatiotemporal control of protein function in cells. Curr Opin Chem Biol.

[25] Carnevale V, Klein ML (2017). Small molecule modulation of voltage gated sodium channels. Curr Opin Struct Biol.

[26] Chen X, Liu D, Zhou D, Si Y, Xu D, Stamatkin CW (2018). Small-molecule CaVα1⋅CaVβ antagonist suppresses neuronal voltage-gated calcium-channel trafficking. Proc Natl Acad Sci U S A.

[27] Wong NML, Wong WW (2018). Engineering a Dual Small Molecule Gated ZAP70 Switch in T Cells. ACS Synth Biol.

[28] Kolos JM, Voll AM, Bauder M, Hausch F (2018). FKBP Ligands—Where We Are and Where to Go?. Front Pharmacol.

[29] Jullien N, Sampieri F, Enjalbert A, Herman J-P (2003). Regulation of Cre recombinase by ligand-induced complementation of inactive fragments. Nucleic Acids Res.

[30] Qudrat A, Truong K (2017). Engineering Synthetic Proteins to Generate Ca2+ Signals in Mammalian Cells. ACS Synth Biol.

[31] Inobe T, Nukina N (2016). Rapamycin-induced oligomer formation system of FRB-FKBP fusion proteins. J Biosci Bioeng.

[32] Zetsche B, Volz SE, Zhang F (2015). A split-Cas9 architecture for inducible genome editing and transcription modulation. Nat Biotechnol.

[33] Bae T, Hur JW, Kim D, Hur JK (2019). Recent trends in CRISPR-Cas system: genome, epigenome, and transcriptome editing and CRISPR delivery systems. Genes Genomics.

[34] Adli M (2018). The CRISPR tool kit for genome editing and beyond. Nat Commun.

[35] Kunz J, Hall MN (1993). Cyclosporin A, FK506 and rapamycin: more than just immunosuppression. Trends Biochem Sci.

[36] Meng L-h, Zheng XFS (2015). Toward rapamycin analog (rapalog)-based precision cancer therapy. Acta Pharmacol Sin.

[37] Bojar D, Scheller L, Hamri GC-E, Xie M, Fussenegger M (2018). Caffeine-inducible gene switches controlling experimental diabetes. Nat Commun.

[38] Kang S, Davidsen K, Gomez-Castillo L, Jiang H, Fu X, Li Z (2019). COMBINES-CID: An Efficient Method for De Novo Engineering of Highly Specific Chemically Induced Protein Dimerization Systems. J Am Chem Soc.

[39] Gossen M, Bujard H (1992). Tight control of gene expression in mammalian cells by tetracycline-responsive promoters. Proc Natl Acad Sci U S A.

[40] Alsaieedi A, Holler A, Velica P, Bendle G, Stauss HJ (2019). Safety and efficacy of Tet-regulated IL-12 expression in cancer-specific T cells. Oncoimmunology.

[41] Sakemura R, Terakura S, Watanabe K, Julamanee J, Takagi E, Miyao K (2016). A Tet-On Inducible System for Controlling CD19-Chimeric Antigen Receptor Expression upon Drug Administration. Cancer Immunol Res.

[42] Gu X, He D, Li C, Wang H, Yang G (2018). Development of Inducible CD19-CAR T Cells with a Tet-On System for Controlled Activity and Enhanced Clinical Safety. Int J Mol Sci.

[43] Long AH, Haso WM, Shern JF, Wanhainen KM, Murgai M, Ingaramo M (2015). 4-1BB costimulation ameliorates T cell exhaustion induced by tonic signaling of chimeric antigen receptors. Nat Med.

[44] Das AT, Tenenbaum L, Berkhout B (2016). Tet-On Systems For Doxycycline-inducible Gene Expression. Curr Gene Ther.

[45] Kumar K, Waldmann H (2019). Nature Inspired Small Molecules for Chemical Biology. Isr J Chem.

[46] Gottesfeld JM, Turner JM, Dervan PB (2000). Chemical approaches to control gene expression. Gene Expr.

[47] Ansari AZ, Mapp AK, Nguyen DH, Dervan PB, Ptashne M (2001). Towards a minimal motif for artificial transcriptional activators. Chem Biol.

[48] Rathnam C, Chueng S-TD, Yang L, Lee K-B (2017). Advanced Gene Manipulation Methods for Stem Cell Theranostics. Theranostics.

[49] Lee DW, Gardner R, Porter DL, Louis CU, Ahmed N, Jensen M (2014). Current concepts in the diagnosis and management of cytokine release syndrome. Blood.

[50] Morgan RA, Yang JC, Kitano M, Dudley ME, Laurencot CM, Rosenberg SA (2010). Case report of a serious adverse event following the administration of T cells transduced with a chimeric antigen receptor recognizing ERBB2. Mol Ther.

[51] Chakravarti D, Caraballo LD, Weinberg BH, Wong WW (2019). Inducible Gene Switches with Memory in Human T Cells for Cellular Immunotherapy. ACS Synth Biol.

[52] Indra AK, Warot X, Brocard J, Bornert JM, Xiao JH, Chambon P (1999). Temporally-controlled site-specific mutagenesis in the basal layer of the epidermis: comparison of the recombinase activity of the tamoxifen-inducible Cre-ER(T) and Cre-ER(T2) recombinases. Nucleic Acids Res.

[53] Nagy A (2000). Cre recombinase: The universal reagent for genome tailoring. Genesis.

[54] Wu CY, Roybal KT, Puchner EM, Onuffer J, Lim WA (2015). Remote control of therapeutic T cells through a small molecule-gated chimeric receptor. Science.

[55] Redelsperger IM, Taldone T, Riedel ER, Lepherd ML, Lipman NS, Wolf FR (2016). Stability of Doxycycline in Feed and Water and Minimal Effective Doses in Tetracycline-Inducible Systems. J Am Assoc Lab Anim Sci.

[56] Weber W, Rimann M, Spielmann M, Keller B, Baba MD-E, Aubel D (2004). Gas-inducible transgene expression in mammalian cells and mice. Nat Biotechnol.

[57] Mancini RJ, Stutts L, Ryu KA, Tom JK, Esser-Kahn AP (2014). Directing the immune system with chemical compounds. ACS Chem Biol.

[58] Juillerat A, Marechal A, Filhol J-M, Valton J, Duclert A, Poirot L (2016). Design of chimeric antigen receptors with integrated controllable transient functions. Sci Rep.

[59] Marro BS, Zak J, Zavareh RB, Teijaro JR, Lairson LL, Oldstone MBA (2019). Discovery of Small Molecules for the Reversal of T Cell Exhaustion. Cell Rep.

[60] Sun S, Hao H, Yang G, Zhang Y, Fu Y (2018). Immunotherapy with CAR-Modified T Cells: Toxicities and Overcoming Strategies. J Immunol Res.

[61] Han X, Bryson PD, Zhao Y, Cinay GE, Li S, Guo Y (2017). Masked Chimeric Antigen Receptor for Tumor-Specific Activation. Mol Ther.

[62] Weniger M, Honselmann KC, Liss AS (2018). The Extracellular Matrix and Pancreatic Cancer: A Complex Relationship. Cancers (Basel).

[63] He L, Zhang Y, Ma G, Tan P, Li Z, Zang S (2015). Near-infrared photoactivatable control of Ca2+ signaling and optogenetic immunomodulation. eLife.

[64] Tischer DK, Weiner OD (2019). Light-based tuning of ligand half-life supports kinetic proofreading model of T cell signaling. eLife.

[65] Yousefi OS, Günther M, Hörner M, Chalupsky J, Wess M, Brandl SM (2019). Optogenetic control shows that kinetic proofreading regulates the activity of the T cell receptor. eLife.

[66] Zhao B, Wang Y, Tan X, Zheng X, Wang F, Ke K (2019). An Optogenetic Controllable T Cell System for Hepatocellular Carcinoma Immunotherapy. Theranostics.

[67] Allen ME, Zhou W, Thangaraj J, Kyriakakis P, Wu Y, Huang Z (2019). An AND-Gated Drug and Photoactivatable Cre-loxP System for Spatiotemporal Control in Cell-Based Therapeutics. ACS Synth Biol.

[68] Macian F (2005). NFAT proteins: key regulators of T-cell development and function. Nat Rev Immunol.

[69] Alexandre MTA, Domratcheva T, Bonetti C, van Wilderen LJGW, van Grondelle R, Groot M-L (2009). Primary reactions of the LOV2 domain of phototropin studied with ultrafast mid-infrared spectroscopy and quantum chemistry. Biophys J.

[70] Che DL, Duan L, Zhang K, Cui B (2015). The Dual Characteristics of Light-Induced Cryptochrome 2, Homo-oligomerization and Heterodimerization, for Optogenetic Manipulation in Mammalian Cells. ACS Synth Biol.

[71] Xu Y, Hyun Y-M, Lim K, Lee H, Cummings RJ, Gerber SA (2014). Optogenetic control of chemokine receptor signal and T-cell migration. Proc Natl Acad Sci U S A.

[72] Shao J, Xue S, Yu G, Yu Y, Yang X, Bai Y (2017). Smartphone-controlled optogenetically engineered cells enable semiautomatic glucose homeostasis in diabetic mice. Sci Transl Med.

[73] Ash C, Dubec M, Donne K, Bashford T (2017). Effect of wavelength and beam width on penetration in light-tissue interaction using computational methods. Lasers Med Sci.

[74] Folcher M, Oesterle S, Zwicky K, Thekkottil T, Heymoz J, Hohmann M (2014). Mind-controlled transgene expression by a wireless-powered optogenetic designer cell implant. Nat Commun.

[75] Gardner L, Deiters A (2012). Light-controlled synthetic gene circuits. Curr Opin Chem Biol.

[76] Cruz FG, Koh JT, Link KH (2000). Light-Activated Gene Expression. J Am Chem Soc.

[77] Coffman RL, Sher A, Seder RA (2010). Vaccine adjuvants: putting innate immunity to work. Immunity.

[78] Sallusto F, Lanzavecchia A (2002). The instructive role of dendritic cells on T-cell responses. Arthritis Res.

[79] Ryu KA, McGonnigal B, Moore T, Kargupta T, Mancini RJ, Esser-Kahn AP (2017). Light Guided In-vivo Activation of Innate Immune Cells with Photocaged TLR 2/6 Agonist. Sci Rep.

[80] Taslimi A, Zoltowski B, Miranda JG, Pathak GP, Hughes RM, Tucker CL (2016). Optimized second-generation CRY2-CIB dimerizers and photoactivatable Cre recombinase. Nat Chem Biol.

[81] Kawano F, Suzuki H, Furuya A, Sato M (2015). Engineered pairs of distinct photoswitches for optogenetic control of cellular proteins. Nat Commun.

[82] Wang X, Chen X, Yang Y (2012). Spatiotemporal control of gene expression by a light-switchable transgene system. Nat Methods.

[83] Tan P, He L, Han G, Zhou Y (2017). Optogenetic Immunomodulation: Shedding Light on Antitumor Immunity. Trends Biotechnol.

[84] Müller K, Engesser R, Timmer J, Zurbriggen MD, Weber W (2014). Orthogonal Optogenetic Triple-Gene Control in Mammalian Cells. ACS Synth Biol.

[85] Polstein LR, Gersbach CA (2012). Light-Inducible Spatiotemporal Control of Gene Activation by Customizable Zinc Finger Transcription Factors. J Am Chem Soc.

[86] Polstein LR, Gersbach CA (2015). A light-inducible CRISPR-Cas9 system for control of endogenous gene activation. Nat Chem Biol.

[87] Zhou XX, Zou X, Chung HK, Gao Y, Liu Y, Qi LS (2018). A Single-Chain Photoswitchable CRISPR-Cas9 Architecture for Light-Inducible Gene Editing and Transcription. ACS Chem Biol.

[88] Furuhata Y, Nihongaki Y, Sato M, Yoshimoto K (2017). Control of Adipogenic Differentiation in Mesenchymal Stem Cells via Endogenous Gene Activation Using CRISPR-Cas9. ACS Synth Biol.

[89] Hsu M-N, Chang Y-H, Truong VA, Lai P-L, Nguyen TKN, Hu Y-C (2019). CRISPR technologies for stem cell engineering and regenerative medicine. Biotechnol Adv.

[90] Ye L, Park JJ, Dong MB, Yang Q, Chow RD, Peng L (2019). In vivo CRISPR screening in CD8 T cells with AAV-Sleeping Beauty hybrid vectors identifies membrane targets for improving immunotherapy for glioblastoma. Nat Biotechnol.

[91] Pan D, Kobayashi A, Jiang P, Ferrari de Andrade L, Tay RE, Luoma AM (2018). A major chromatin regulator determines resistance of tumor cells to T cell-mediated killing. Science.

[92] Ruggiero E, Alonso-de Castro S, Habtemariam A, Salassa L (2016). Upconverting nanoparticles for the near infrared photoactivation of transition metal complexes: new opportunities and challenges in medicinal inorganic photochemistry. Dalton Trans.

[93] Fry HC, Lehmann A, Sinks LE, Asselberghs I, Tronin A, Krishnan V (2013). Computational de novo design and characterization of a protein that selectively binds a highly hyperpolarizable abiological chromophore. J Am Chem Soc.

[94] Torchilin VP (2014). Multifunctional, stimuli-sensitive nanoparticulate systems for drug delivery. Nat Rev Drug Discov.

[95] Wust P, Hildebrandt B, Sreenivasa G, Rau B, Gellermann J, Riess H (2002). Hyperthermia in combined treatment of cancer. Lancet Oncol.

[96] Hamazoe R, Maeta M, Kaibara N (1994). Intraperitoneal thermochemotherapy for prevention of peritoneal recurrence of gastric cancer. Final results of a randomized controlled study. Cancer.

[97] Zhu L, Altman MB, Laszlo A, Straube W, Zoberi I, Hallahan DE (2019). Ultrasound Hyperthermia Technology for Radiosensitization. Ultrasound Med Biol.

[98] Li W, Hou W, Guo X, Luo L, Li Q, Zhu C (2018). Temperature-controlled, phase-transition ultrasound imaging-guided photothermal-chemotherapy triggered by NIR light. Theranostics.

[99] Brace C (2011). Thermal tumor ablation in clinical use. IEEE Pulse.

[100] Partanen A, Yarmolenko PS, Viitala A, Appanaboyina S, Haemmerich D, Ranjan A (2012). Mild hyperthermia with magnetic resonance-guided high-intensity focused ultrasound for applications in drug delivery. Int J Hyperthermia.

[101] Chang D, Lim M, Goos JACM, Qiao R, Ng YY, Mansfeld FM (2018). Biologically Targeted Magnetic Hyperthermia: Potential and Limitations. Front Pharmacol.

[102] Zou J, Guo Y, Guettouche T, Smith DF, Voellmy R (1998). Repression of Heat Shock Transcription Factor HSF1 Activation by HSP90 (HSP90 Complex) that Forms a Stress-Sensitive Complex with HSF1. Cell.

[103] Lindquist S (1986). The Heat-Shock Response. Annual Review of Biochemistry.

[104] Kortmann J, Narberhaus F (2012). Bacterial RNA thermometers: molecular zippers and switches. Nat Rev Microbiol.

[105] Piraner DI, Abedi MH, Moser BA, Lee-Gosselin A, Shapiro MG (2016). Tunable thermal bioswitches for in vivo control of microbial therapeutics. Nat Chem Biol.

[106] Richter F, Fonfara I, Bouazza B, Schumacher CH, Bratovič M, Charpentier E (2016). Engineering of temperature- and light-switchable Cas9 variants. Nucleic Acids Res.

[107] Stanley SA, Gagner JE, Damanpour S, Yoshida M, Dordick JS, Friedman JM (2012). Radio-Wave Heating of Iron Oxide Nanoparticles Can Regulate Plasma Glucose in Mice. Science.

[108] Bai P, Liu Y, Xue S, Hamri GC-E, Saxena P, Ye H (2019). A fully human transgene switch to regulate therapeutic protein production by cooling sensation. Nat Med.

[109] Jiang Y, Carvalho-de-Souza JL, Wong RCS, Luo Z, Isheim D, Zuo X (2016). Heterogeneous silicon mesostructures for lipid-supported bioelectric interfaces. Nat Mater.

[110] Vekris A, Maurange C, Moonen C, Mazurier F, De Verneuil H, Canioni P (2000). Control of transgene expression using local hyperthermia in combination with a heat-sensitive promoter. J Gene Med.

[111] Vilaboa N, Fenna M, Munson J, Roberts SM, Voellmy R (2005). Novel Gene Switches for Targeted and Timed Expression of Proteins of Interest. Mol Ther.

[112] Huang Q, Hu JK, Lohr F, Zhang L, Braun R, Lanzen J (2000). Heat-induced Gene Expression as a Novel Targeted Cancer Gene Therapy Strategy. Cancer Res.

[113] Tang Q-s, Zhang D-s, Cong X-m, Wan M-l, Jin L-q (2008). Using thermal energy produced by irradiation of Mn-Zn ferrite magnetic nanoparticles (MZF-NPs) for heat-inducible gene expression. Biomaterials.

[114] Deckers R, Quesson B, Arsaut J, Eimer S, Couillaud F, Moonen CTW (2009). Image-guided, noninvasive, spatiotemporal control of gene expression. Proc Natl Acad Sci U S A.

[115] Siddiqui F, Li C-Y, LaRue SM, Poulson JM, Avery PR, Pruitt AF (2007). A phase I trial of hyperthermia-induced interleukin-12 gene therapy in spontaneously arising feline soft tissue sarcomas. Mol Cancer Ther.

[116] Andersson HA, Kim Y-S, O'Neill BE, Shi Z-Z, Serda RE (2014). HSP70 promoter-driven activation of gene expression for immunotherapy using gold nanorods and near infrared light. Vaccines (Basel).

[117] Miller IC, Gamboa Castro M, Maenza J, Weis JP, Kwong GA (2018). Remote Control of Mammalian Cells with Heat-Triggered Gene Switches and Photothermal Pulse Trains. ACS Synth Biol.

[118] Fogar P, Navaglia F, Basso D, Zambon CF, Moserle L, Indraccolo S (2010). Heat-induced transcription of diphtheria toxin A or its variants, CRM176 and CRM197: implications for pancreatic cancer gene therapy. Cancer Gene Ther.

[119] Ramirez VP, Stamatis M, Shmukler A, Aneskievich BJ (2015). Basal and stress-inducible expression of HSPA6 in human keratinocytes is regulated by negative and positive promoter regions. Cell Stress Chaperones.

[120] Shamovsky I, Ivannikov M, Kandel ES, Gershon D, Nudler E (2006). RNA-mediated response to heat shock in mammalian cells. Nature.

[121] Sen S, Apurva D, Satija R, Siegal D, Murray RM (2017). Design of a Toolbox of RNA Thermometers. ACS Synth Biol.

[122] Neupert J, Bock R (2009). Designing and using synthetic RNA thermometers for temperature-controlled gene expression in bacteria. Nat Protoc.

[123] Gamboa L, Phung EV, Li H, Meyers JP, Hart AC, Miller IC Heat-Triggered Remote Control of CRISPR-dCas9 for Tunable Transcriptional Modulation. ACS Chem Biol, in press.

[124] Lasek W, Zagożdżon R, Jakobisiak M (2014). Interleukin 12: still a promising candidate for tumor immunotherapy?. Cancer Immunol Immunother.

[125] Richter K, Haslbeck M, Buchner J (2010). The Heat Shock Response: Life on the Verge of Death. Mol Cell.

[126] Ortner V, Ludwig A, Riegel E, Dunzinger S, Czerny T (2015). An artificial HSE promoter for efficient and selective detection of heat shock pathway activity. Cell Stress Chaperones.

[127] Elias WJ, Lipsman N, Ondo WG, Ghanouni P, Kim YG, Lee W (2016). A Randomized Trial of Focused Ultrasound Thalamotomy for Essential Tremor. N Engl J Med.

[128] Nuñez JK, Harrington LB, Doudna JA (2016). Chemical and Biophysical Modulation of Cas9 for Tunable Genome Engineering. ACS Chem Biol.

[129] Pan Y, Yoon S, Sun J, Huang Z, Lee C, Allen M (2018). Mechanogenetics for the remote and noninvasive control of cancer immunotherapy. Proc Natl Acad Sci U S A.

[130] Seo D, Southard KM, Kim J-w, Lee HJ, Farlow J, Lee J-u (2016). A Mechanogenetic Toolkit for Interrogating Cell Signaling in Space and Time. Cell.

[131] Pan Y, Yoon S, Zhu L, Wang Y (2018). Acoustic mechanogenetics. Curr Opin Biomed Eng.

[132] Zhu H, Zhang L, Tong S, Lee CM, Deshmukh H, Bao G (2019). Spatial control of in vivo CRISPR-Cas9 genome editing via nanomagnets. Nat Biomed Eng.

[133] Coste B, Mathur J, Schmidt M, Earley TJ, Ranade S, Petrus MJ (2010). Piezo1 and Piezo2 are essential components of distinct mechanically activated cation channels. Science.

[134] Steinbuck MP, Winandy S (2018). A Review of Notch Processing With New Insights Into Ligand-Independent Notch Signaling in T-Cells. Front Immunol.

[135] Wheeler MA, Smith CJ, Ottolini M, Barker BS, Purohit AM, Grippo RM (2016). Genetically targeted magnetic control of the nervous system. Nat Neurosci.

[136] Chen R, Romero G, Christiansen MG, Mohr A, Anikeeva P (2015). Wireless magnetothermal deep brain stimulation. Science.

[137] Nimpf S, Keays DA (2017). Is magnetogenetics the new optogenetics?. EMBO J.

[138] Long X, Ye J, Zhao D, Zhang S-J (2015). Magnetogenetics: remote non-invasive magnetic activation of neuronal activity with a magnetoreceptor. Sci Bull (Beijing).

[139] Morsut L, Roybal KT, Xiong X, Gordley RM, Coyle SM, Thomson M (2016). Engineering Customized Cell Sensing and Response Behaviors Using Synthetic Notch Receptors. Cell.

[140] Chang ZL, Lorenzini MH, Chen X, Tran U, Bangayan NJ, Chen YY (2018). Rewiring T-cell responses to soluble factors with chimeric antigen receptors. Nat Chem Biol.

[141] Tang L, Zheng Y, Melo MB, Mabardi L, Castaño AP, Xie Y-Q (2018). Enhancing T cell therapy through TCR-signaling-responsive nanoparticle drug delivery. Nat Biotechnol.

[142] Yarchoan M, Johnson BA 3rd, Lutz ER, Laheru DA, Jaffee EM (2017). Targeting neoantigens to augment antitumour immunity. Nature reviews Cancer.

[143] Li H, Zhao Y (2017). Increasing the safety and efficacy of chimeric antigen receptor T cell therapy. Protein Cell.

[144] Ruella M, Barrett DM, Kenderian SS, Shestova O, Hofmann TJ, Perazzelli J (2016). Dual CD19 and CD123 targeting prevents antigen-loss relapses after CD19-directed immunotherapies. J Clin Invest.

[145] Hegde M, Mukherjee M, Grada Z, Pignata A, Landi D, Navai SA (2016). Tandem CAR T cells targeting HER2 and IL13Rα2 mitigate tumor antigen escape. J Clin Invest.

[146] Kloss CC, Condomines M, Cartellieri M, Bachmann M, Sadelain M (2012). Combinatorial antigen recognition with balanced signaling promotes selective tumor eradication by engineered T cells. Nat Biotechnol.

[147] Fedorov VD, Themeli M, Sadelain M (2013). PD-1- and CTLA-4-Based Inhibitory Chimeric Antigen Receptors (iCARs) Divert Off-Target Immunotherapy Responses. Sci Transl Med.

[148] Aalipour A, Chuang H-Y, Murty S, D'Souza AL, Park S-m, Gulati GS (2019). Engineered immune cells as highly sensitive cancer diagnostics. Nat Biotechnol.

[149] Kato Y, Ozawa S, Miyamoto C, Maehata Y, Suzuki A, Maeda T (2013). Acidic extracellular microenvironment and cancer. Cancer Cell Int.

[150] Wang N, Dong C-R, Jiang R, Tang C, Yang L, Jiang Q-F (2013). Overexpression of HIF-1α, metallothionein and SLUG is associated with high TNM stage and lymph node metastasis in papillary thyroid carcinoma. Int J Clin Exp Pathol.

[151] Liu N, Sun Y, Zhao N, Chen L (2014). Role of hypoxia-inducible factor-1α and survivin in oxygen-induced retinopathy in mice. Int J Clin Exp Pathol.

[152] Yin X, Shin H-D, Li J, Du G, Liu L, Chen J (2017). Pgas, a Low-pH-Induced Promoter, as a Tool for Dynamic Control of Gene Expression for Metabolic Engineering of Aspergillus niger. Appl Environ Microbiol.

[153] Hou Q, He Q, Liu G, Lu X, Zong H, Chen W (2019). Identification and application of novel low pH-inducible promoters for lactic acid production in the tolerant yeast Candida glycerinogenes. J Biosci Bioeng.

[154] Shen Y, Tang H, Radosz M, Van Kirk E, Murdoch WJ, Jain KK (2008). pH-Responsive Nanoparticles for Cancer Drug Delivery. Drug Delivery Systems.

[155] Liu Y, Pan Y, Cao W, Xia F, Liu B, Niu J (2019). A tumor microenvironment responsive biodegradable CaCO(3)/MnO(2)- based nanoplatform for the enhanced photodynamic therapy and improved PD-L1 immunotherapy. Theranostics.

[156] Madhusudhan A, Reddy GB, Venkatesham M, Veerabhadram G, Kumar DA, Natarajan S (2014). Efficient pH dependent drug delivery to target cancer cells by gold nanoparticles capped with carboxymethyl chitosan. Int J Mol Sci.

[157] Uthaman S, Huh KM, Park I-K (2018). Tumor microenvironment-responsive nanoparticles for cancer theragnostic applications. Biomater Res.

[158] Roma-Rodrigues C, Pombo I, Raposo L, Pedrosa P, Fernandes AR, Baptista PV (2019). Nanotheranostics Targeting the Tumor Microenvironment. Front Bioeng Biotechnol.

[159] Calcinotto A, Filipazzi P, Grioni M, Iero M, De Milito A, Ricupito A (2012). Modulation of microenvironment acidity reverses anergy in human and murine tumor-infiltrating T lymphocytes. Cancer Res.

[160] Berahovich R, Liu X, Zhou H, Tsadik E, Xu S, Golubovskaya V (2019). Hypoxia Selectively Impairs CAR-T Cells In Vitro. Cancers (Basel).

[161] Thomas DA, Massagué J (2005). TGF-β directly targets cytotoxic T cell functions during tumor evasion of immune surveillance. Cancer Cell.

[162] Martín-Saavedra FM, Wilson CG, Voellmy R, Vilaboa N, Franceschi RT (2013). Spatiotemporal control of vascular endothelial growth factor expression using a heat-shock-activated, rapamycin-dependent gene switch. Hum Gene Ther Methods.

[163] Chen X, Li T, Wang X, Du Z, Liu R, Yang Y (2015). Synthetic dual-input mammalian genetic circuits enable tunable and stringent transcription control by chemical and light. Nucleic Acids Res.

[164] Liu L, Huang W, Huang J-D (2017). Synthetic circuits that process multiple light and chemical signal inputs. BMC Syst Biol.

[165] Kristianto J, Johnson MG, Zastrow RK, Radcliff AB, Blank RD (2017). Spontaneous recombinase activity of Cre-ERT2 in vivo. Transgenic Res.

[166] Coley WB (1891). II. Contribution to the Knowledge of Sarcoma. Ann Surg.

[167] Forbes NS (2010). Engineering the perfect (bacterial) cancer therapy. Nat Rev Cancer.

[168] Zheng JH, Nguyen VH, Jiang SN, Park SH, Tan W, Hong SH (2017). Two-step enhanced cancer immunotherapy with engineered Salmonella typhimurium secreting heterologous flagellin. Sci Transl Med.

[169] Jiang SN, Phan TX, Nam TK, Nguyen VH, Kim HS, Bom HS (2010). Inhibition of tumor growth and metastasis by a combination of Escherichia coli-mediated cytolytic therapy and radiotherapy. Mol Ther.

[170] Danino T, Prindle A, Kwong GA, Skalak M, Li H, Allen K (2015). Programmable probiotics for detection of cancer in urine. Sci Transl Med.

[171] Prindle A, Samayoa P, Razinkov I, Danino T, Tsimring LS, Hasty J (2011). A sensing array of radically coupled genetic 'biopixels'. Nature.

[172] Din MO, Danino T, Prindle A, Skalak M, Selimkhanov J, Allen K (2016). Synchronized cycles of bacterial lysis for in vivo delivery. Nature.

[173] Chowdhury S, Castro S, Coker C, Hinchliffe TE, Arpaia N, Danino T (2019). Programmable bacteria induce durable tumor regression and systemic antitumor immunity. Nat Med.

[174] Dahotre SN, Chang YM, Wieland A, Stammen SR, Kwong GA (2018). Individually addressable and dynamic DNA gates for multiplexed cell sorting. Proc Natl Acad Sci U S A.

[175] Adleman LM (1994). Molecular computation of solutions to combinatorial problems. Science.

[176] Pei R, Matamoros E, Liu M, Stefanovic D, Stojanovic MN (2010). Training a molecular automaton to play a game. Nat Nanotechnol.

[177] Dahotre SN, Chang YM, Romanov AM, Kwong GA (2019). DNA-Barcoded pMHC Tetramers for Detection of Single Antigen-Specific T Cells by Digital PCR. Anal Chem.

[178] Dudani JS, Warren AD, Bhatia SN (2018). Harnessing Protease Activity to Improve Cancer Care.

[179] Kwong GA, Dudani JS, Carrodeguas E, Mazumdar EV, Zekavat SM, Bhatia SN (2015). Mathematical framework for activity-based cancer biomarkers. Proc Natl Acad Sci U S A.

[180] Kwong GA, von Maltzahn G, Murugappan G, Abudayyeh O, Mo S, Papayannopoulos IA (2013). Mass-encoded synthetic biomarkers for multiplexed urinary monitoring of disease. Nat Biotechnol.

[181] Mac QD, Mathews DV, Kahla JA, Stoffers CM, Delmas OM, Holt BA (2019). Non-invasive early detection of acute transplant rejection via nanosensors of granzyme B activity. Nat Biomed Eng.

[182] Larimer BM, Wehrenberg-Klee E, Dubois F, Mehta A, Kalomeris T, Flaherty K (2017). Granzyme B PET Imaging as a Predictive Biomarker of Immunotherapy Response. Cancer Res.

[183] Krekorian M, Fruhwirth GO, Srinivas M, Figdor CG, Heskamp S, Witney TH (2019). Imaging of T-cells and their responses during anti-cancer immunotherapy. Theranostics.

[184] Gao XJ, Chong LS, Kim MS, Elowitz MB (2018). Programmable protein circuits in living cells. Science.

[185] Holt BA, Kwong GA (2019). Proteases as Biological Bits for Programmable Medicine.

[186] Hobeika E, Dautzenberg M, Levit-Zerdoun E, Pelanda R, Reth M (2018). Conditional Selection of B Cells in Mice With an Inducible B Cell Development. Front Immunol.

[187] Oide M, Okajima K, Kashojiya S, Takayama Y, Oroguchi T, Hikima T (2016). Blue Light-excited Light-Oxygen-Voltage-sensing Domain 2 (LOV2) Triggers a Rearrangement of the Kinase Domain to Induce Phosphorylation Activity in Arabidopsis Phototropin1. J Biol Chem.

[188] Huse M (2010). Photochemical approaches to T-cell activation. Immunology.

